# Anti-inflammatory Diet Index and Bladder Cancer Risk by Stage: A 22-Year Prospective Swedish Cohort Study (1998–2020)

**DOI:** 10.1158/1055-9965.EPI-25-1733

**Published:** 2026-03-31

**Authors:** Samira Prado, Henrik Ugge, Anders Vikerfors, Katja Fall, Tahir Taj

**Affiliations:** 1 Nutrition-Gut-Brain Interactions Research Centre, School of Medical Sciences, Faculty of Medicine and Health, Örebro University, Örebro, Sweden.; 2 Faculty of Business, Science and Engineering, School of Science and Technology, Örebro University, Örebro, Sweden.; 3 Food and Health Center, Örebro University, Örebro, Sweden.; 4 Department of Urology, Faculty of Medicine and Health, Örebro University, Örebro, Sweden.; 5 Clinical Epidemiology and Biostatistics, School of Medical Sciences, Faculty of Medicine and Health, Örebro University, Örebro, Sweden.

## Abstract

**Background:**

Dietary patterns influencing systemic levels of inflammation have been proposed and investigated as possible determinants of cancer risk. We evaluated the association between the anti-inflammatory potential of diet and the risk of bladder cancer.

**Methods:**

The anti-inflammatory diet index (AIDI), composed of 16 food groups (11 anti- and 5 pro-inflammatory), was used to score dietary patterns in *N* = 79,292 individuals derived from the Cohort of Swedish Men (established in 1997) and the Swedish Mammography Cohort (established in 1987). Dietary information was collected in 1997 and 2009; repeated-measures analyses used a cumulative-average AIDI. Incident bladder cancer cases were identified from the Swedish Cancer Register using International Classification of Diseases, 10th Revision code C67, and a baseline study questionnaire was used to assess covariates. We estimated multivariable hazard ratios (HR) across AIDI quartiles using Cox models.

**Results:**

After a 22-year follow-up, 1,165 individuals were diagnosed with bladder cancer, of whom 249 had non–muscle invasive bladder cancer (NMIBC), 201 had muscle-invasive bladder cancer (MIBC), and 715 had unknown stage. In repeated-measures analyses, the highest anti-inflammatory quartile (Q4) was associated with lower bladder cancer risk compared with the lowest quartile [Q1; HR, 0.74; 95% confidence interval (CI), 0.61–0.89]. Stratifying for tumor stage, there was a clear association between AIDI score and MIBC (HR, 0.35; 95% CI, 0.22–0.57) but not for NMIBC (HR, 0.86; 95% CI, 0.57–1.28).

**Conclusions:**

An anti-inflammatory dietary pattern was associated with lower bladder cancer risk, with the clearest association for MIBC.

**Impact:**

These findings support the role of dietary inflammation in bladder cancer etiology and suggest that promoting anti-inflammatory dietary patterns could contribute to cancer prevention strategies.

## Introduction

Bladder cancer is the 10th most diagnosed cancer globally, accounting for 573,000 new cases and 213,000 deaths annually (1). It is more common among men than women (ratio 4:1), and the dominating histologic subtype is urothelial carcinoma (2). The highest incidence is in Europe, North America, Western Asia, and North Africa, which is considered to reflect the distribution of important risk factors (2). Tobacco smoking is the most significant risk factor, estimated to contribute to about 50% of bladder cancer cases (2). Other risk factors include *Schistosoma haematobium* infection, particularly in Northern and Sub-Saharan Africa, commonly associated with squamous cell carcinoma of the bladder, and environmental or occupational exposure to various carcinogens (1, 3). Diagnosis and classification of bladder cancer rely on assessing the depth of tumor invasion, which are categorized as either non–muscle invasive bladder cancer (NMIBC), confined to the mucosa or submucosa, or muscle-invasive bladder cancer (MIBC), with the tumor invading into or beyond the muscularis propria (4).

Chronic inflammation plays a complex role in bladder cancer development and progression (5). Among modifiable risk factors, diet has emerged as an important determinant of low-grade systemic inflammation. The anti-inflammatory diet index (AIDI) has been developed to assess the inflammatory potential of dietary patterns and has been shown to predict low-grade systemic inflammation, with higher scores correlating with lower C-reactive protein (CRP) levels (6).

Several studies have reported a positive association between bladder cancer risk and dietary patterns characterized by high intake of alcohol, coffee, organ meats, and a typical Western diet (7–10). In contrast, an inverse association has been observed for diets rich in dairy products, fruits, vegetables, whole grains, and fibers, as well as adherence to Mediterranean diet pattern (11–16). Diets with high pro-inflammatory potential, rich in energy, carbohydrate, protein, fat, cholesterol, vitamin B12, and iron, have been associated with increased bladder cancer risk (17).

These findings suggest that adherence to a diet with high anti-inflammatory potential may reduce the risk of bladder cancer. No large prospective study has evaluated anti-inflammatory dietary patterns by bladder cancer stage, and we address this gap in this study. Therefore, our aim was to evaluate the association between the anti-inflammatory potential of diet and the risk of bladder cancer in a 22-year prospective Swedish cohort study (1998–2020).

## Materials and Methods

### Study population

The study drew data from two cohorts: the Swedish Mammography Cohort (SMC) and the Cohort of Swedish Men (COSM). The SMC was established in 1987 and included all women born between 1914 and 1948 who lived in Västmanland and Uppsala counties, part of Sweden’s central healthcare region. The COSM, created in 1997, consisted of all men born between 1918 and 1952 residing in Västmanland and Örebro counties, also within the central healthcare region of Sweden. Researchers can access data from these cohorts through the Swedish Infrastructure for Medical Population-Based Life-Course and Environmental Research (SIMPLER; https://www.simpler4health.se). Food frequency questionnaires were administered in 1997 and again in 2009 to investigate exhaustive information on the participants’ dietary habits. In the cumulative-average approach, participants contributed AIDI based on the baseline food frequency questionnaire (FFQ; 1997) from the start of follow-up until the completion of the second FFQ (2009). From 2009 onward, AIDI was updated to the cumulative average of the 1997 and 2009 assessments for participants with dietary data from both FFQs. The components of AIDI are described in Supplementary Table S1.

Participants in the study were linked to the Swedish Cancer Register, the Swedish Cause of Death Register, and the Swedish Population Register using their unique 12-digit personal identification numbers. Details about the date and cause of death were sourced from the Swedish Cause of Death Register. In Sweden, all deaths are registered within 30 days, with a specific cause of death reported for 96% of cases. Information on emigration from Sweden during the study period was obtained from the Swedish Population Register. From a total population of 83,165 participants, we excluded 3,873 (4.6%) participants because of missing exposure or covariates, leaving 79,292 for analysis; of whom 36,101 were women and 43,191 were men. At baseline, participants were on average 62 years of age across quartiles of the AIDI and cancer free.

The study was approved by the Swedish Ethical Review Authority (Dnr 2021-03094). All procedures involving human participants were conducted in accordance with recognized ethical principles, including the Declaration of Helsinki, and applicable national regulations. All participants provided written informed consent at cohort enrollment. Data were deidentified prior to analysis to protect participant confidentiality.

### The AIDI

Dietary assessment was conducted using a 96-item FFQ tailored to capture Swedish dietary habits and measure food consumption over the year before. This questionnaire was administered at baseline in 1997 (fall) and again in 2009 (summer). Participants were asked about average intake frequency for each food item by choosing from eight response options, ranging from never to more than three times daily. Of 30,134 women contacted in 2009, 84% responded, and of 29,068 men contacted in the same year, 90% responded (18).

Daily energy intake was calculated for each individual by combining reported consumption frequency with the energy content of age-specific portion sizes, based on nutrient composition data from the Swedish Food Administration Database. The detailed method of developing the AIDI has been described in earlier publications (6). Briefly, AIDI classifies foods based on their proposed anti- or pro-inflammatory effects. It was based on 16 predefined food items or food groups according to their reported associations with serum high-sensitive CRP, a known inflammatory marker. Among these, 11 components were considered anti-inflammatory and 5 pro-inflammatory. The anti-inflammatory components were total fruits and vegetables (≥6 servings/day), tea (≥3 servings/day), coffee (≥2 servings/day), wholegrain bread (≥2 servings/day), breakfast cereal (≥1 serving/day), low-fat cheese (≥1 serving/day), olive and canola oil (>0 servings/day), chocolate (≥1 serving/day), nuts (≥2 servings/week), red wine (2–7 servings/week), and beer (2–14 servings/week). The pro-inflammatory components were unprocessed red meat (≤0.5 servings/day), processed red meat (≤0.5 servings/day), offal (no consumption), chips (no consumption), and soft drinks (no consumption). For each component, participants were assigned 1 point when the predefined cutoff was achieved and 0 points otherwise, given a total score ranging from 0 to 16. A score of 16 on the AIDI represents dietary habits with the highest anti-inflammatory potential, whereas a score of 0 reflects those with the lowest. The AIDI scale was validated using empirical data from a subgroup of the SMC, involving 3,503 women between 56 and 74 years of age (6), and has since been used in multiple studies conducted in Sweden (19–21).

### Covariates

Participants provided detailed information about their overall health, including smoking habits, height, weight, and comorbidities like diabetes, hypertension, and hypercholesterolemia. Data on regular medication use were also gathered, covering both prescribed drugs and over-the-counter medications, such as vitamins and dietary supplements. Additionally, the questionnaire included socioeconomic and demographic factors, asking participants to report their highest level of education (primary, secondary, or university), employment status (full time, part time, unemployed, student, disability pension, or retired), and civil status (single, married/cohabiting, divorced, or widowed).

### Bladder cancer incidence

Bladder cancer cases were identified through the Swedish Cancer Register. Individuals with any previous cancer diagnosis (except for nonmelanoma skin cancer) prior to baseline were excluded from the analysis. In Sweden, all newly diagnosed tumors must be reported to the national Swedish Cancer Register, in accordance with the regulations set by the National Board of Health and Welfare (SOSFS2006:15). The register is recognized for its high validity in recording data for most common solid tumors and meets the quality criteria established by the International Agency for Research on Cancer (22). Bladder cancer cases were identified using the International Classification of Diseases, 10th Revision code C67. For analyses based on bladder cancer stage, we categorized cases as either NMIBC (Tis, Ta, or T1 with N0 and M0) or MIBC (≥T2 or ≥ N1 or ≥ M1), according to the tumor–node–metastasis (TNM) classification provided by the Swedish Cancer Register (23). Notably, information on TNM status was available only for cases diagnosed after 2004.

### Statistical analysis

We applied Cox proportional hazards regression with attained age as the underlying time scale to evaluate the associations between AIDI and bladder cancer risk. Follow-up began on January 1, 1998, and continued until the first occurrence of any cancer diagnosis other than melanoma skin cancer, death, emigration, or the end of the follow-up in December 2020, whichever came first. Potential confounders were identified *a priori* based on the established bladder cancer risk factors reported in the literature and the availability of relevant variables in the dataset. Adjustment for potential confounders was performed in three stages. Model 1 accounted for age through the time scale and included sex. Model 2 additionally incorporated individual-level covariates, including smoking exposure as pack-years, body mass index (BMI), employment status (full-time employment, part-time employment, unemployment, studying, disability pension, or retirement), civil status (single, married/cohabiting, divorced, or widower), education level (primary, secondary, or university), and total energy intake, which was mean-centered within sex. Model 3, considered the main model, was further adjusted for comorbidities and family history, including diabetes, hypertension, and family history of cancer (all coded yes/no). Complete-case analysis was used. Therefore, participants with missing exposure data or incomplete information on any covariate were excluded.

Hazard ratios (HR) and 95% confidence intervals (CI) for overall bladder cancer and stage-specific bladder cancer were estimated using Cox regression. AIDI was categorized into quartiles, with the lowest category, representing the most pro-inflammatory dietary pattern, used as the reference group: ≤5 (Q1: range, 0–4; median, 4), 5 (Q2), 6 (Q3), and ≥7 (Q4: range, 7–13; median, 8). In secondary analyses focusing separately on the anti- and pro-inflammatory score components, the score ranges differed because of changes in the observed distribution and minimum/maximum values. Category cutpoints were derived from the distribution in the total study and then applied to men and women. Two principal exposure analyses were conducted: first, using the 1997 baseline FFQ as the exposure measure and second, using a cumulative-average AIDI derived from the 1997 and 2009 FFQs among participants who responded to the 2009 survey. The primary analysis covered the full follow-up period from 1998 to 2020. Tests for trend were based on Cox regression models in which ordinal exposure categories were entered as a continuous variable.

Secondary analyses were undertaken to assess whether the observed associations were predominantly attributed to the pro-inflammatory or anti-inflammatory components of the AIDI. For this purpose, the main analytic model was additionally adjusted, separately for the pro-inflammatory and anti-inflammatory food components, allowing the contribution of the remaining score components to be examined independently. We also performed sex-stratified analyses to investigate possible sex-specific associations between AIDI and bladder cancer risk. All analyses were conducted in R, version 4.1.1 (R Foundation for Statistical Computing).

## Results

The baseline characteristics of Swedish men and women by quartiles of the AIDI, with scores ranging from 0 to 13, are reported in Table 1. The study included 79,292 participants divided into four quartiles. Higher AIDI scores correlated with a greater proportion of women (39% to 53%) and a higher level of education, with university attendance increasing from 12% to 26%. Participants with higher AIDI scores also had lower smoking pack-years (11 to 8), lower BMI (25.9 to 24.9), and were more likely to be married or cohabiting (81%–85%). Employment status showed slight increases in full-time and part-time employment and a decrease in unemployment and disability pension. Hypertension and diabetes diagnoses were less common in higher AIDI quartiles. Energy intake decreased slightly from 2,278 to 2,217 kcal/day. Daily consumption of fruits, vegetables, wholegrain bread, breakfast cereals, tea, and nuts increased with higher AIDI scores, whereas consumption of processed meats and soft drinks decreased. Table 2 illustrates the association between the AIDI and bladder cancer risk. Overall, a higher AIDI score, indicating a more anti-inflammatory diet, was associated with a reduced risk of bladder cancer, especially for MIBC. For overall bladder cancer, the highest quartile of AIDI (Q4, score 8–13) demonstrated a significant inverse relationship, with HRs of 0.81 (95% CI, 0.70–0.95) in the baseline analysis and 0.67 (95% CI, 0.56–0.81) in the repeated-measures analysis, indicating an enhanced protective effect over time. Specifically, for MIBC, the risk reduction was most pronounced in the highest quartile, showing HRs of 0.55 (95% CI, 0.36–0.82) at baseline and 0.34 (95% CI, 0.21–0.54) in the repeated-measures analysis, both with significant *P* values for trend. Conversely, no statistically significant associations were observed for NMIBC in the repeated-measures analysis. Higher AIDI score was associated with a lower risk of total bladder cancer and MIBC in both women and men, particularly in the repeated-measures analyses (Supplementary Tables S2 and S3).

**Table 1.** Descriptive baseline characteristics of Swedish men and women by quartiles of the AIDI (maximum score = 13).

Characteristic	Quartiles of AIDI			
	0–4	5	6	7–13
Number of participants	25,023	16,446	15,698	22,125
Cases	411/24,612	262/16,184	218/15,480	274/21,851

Characteristic	Quartiles of AIDI				*P* overall
	0–4	5	6	7–13	
Years of follow-up	17.7 (6.8)	17.9 (6.7)	18 (6.6)	18.7 (6.3)	0.001
Sex	​	​	​	​	<0.001
Female	9,777 (39%)	7,144 (43%)	7,379 (47%)	11,801 (53%)	​
Male	15,246 (61%)	9,302	8,319 (53%)	10,324 (47%)	​
Mean age at baseline	61.5 (±9.6)	62	62.3 (±9.6)	61.8 (±9.4)	0.001
Education	​	​	​	​	0.001
Primary	10,743 (43%)	6,673 (41%)	5,908 (38%)	6,491 (29%)	​
Secondary	11,363 (45%)	7,418	6,981 (45%)	9,824 (44%)	​
University	2,917 (12%)	2,355 (14%)	2,809 (18%)	5,810 (26%)	​
Smoking, pack-years	11 (±14.9)	10.1 (±14.1)	9.1 (±13.3)	8 (±12.2)	<0.001
BMI	25.9 (±3.9)	25.5	25.3 (±3.6)	24.9 (±3.4)	<0.001
Civil status^a^	​	​	​	​	<0.001
Single	1,148	608	353	535	​
Married/cohabiting	12,321 (81%)	7,670 (83%)	6,876 (83%)	8,784 (85%)	​
Divorced	1,155	633	551	634	​
Widowed	564	353	325	339	​
Employment	​	​	​	​	<0.001
Full time	10,161 (41%)	6,519 (40%)	6,251 (40%)	9,472 (43%)	​
Part time	2,071	1,495	1,418	2,266 (10%)	​
Unemployed	1,212	697	559	700	​
Studying	3,980 (16%)	2,886 (18%)	3,030 (19%)	4,259 (19%)	​
Disability pension	1,851	1,065	907	1,141	​
Retired	5,748 (23%)	3,784 (23%)	3,533 (23%)	4,287 (19%)	​
Ever diagnosed with hypertension	​	​	​	​	<0.001
No	22,198 (89%)	14,731 (90%)	14,199 (91%)	20,246 (92%)	​
Yes	2,825 (11%)	1,715 (10%)	1,499 (9%)	1,879	​
Ever diagnosed with diabetes	​	​	​	​	<0.001
No	24,241 (97%)	15,980 (97%)	15,313 (97%)	21,683 (98%)	​
Yes	782	466	385	442	​
Family history of cancer	​	​	​	​	0.101
No	13,841 (55%)	9,071 (55%)	8,660 (55%)	12,004 (54%)	​
Yes	11,182 (45%)	7,375 (45%)	7,038 (45%)	10,121 (46%)	​
Energy intake, kcal per day	2,278 (±939)	2,255 (±928)	2,205 (±880)	2,217 (±832)	<0.001
Food consumption per day	​	​	​	​	​
Fruits and vegetables^b^	3.6 (±2.1)	4.1 (±2.5)	4.4 (±2.7)	5.4 (±3.1)	<0.001
Wholegrain bread^b^	3.9 (±3.5)	4.3 (±3.4)	4.5 (±3.4)	4.8 (±3.4)	<0.001
Breakfast cereal^b^	0.5 (±0.6)	0.7 (±0.7)	0.8 (±0.7)	1 (±0.7)	<0.001
Unprocessed meat^c^	0.5 (±0.4)	0.5 (±0.4)	0.4 (±0.3)	0.4 (±0.3)	<0.001
Processed meat^c^	1 (±0.7)	0.8 (±0.7)	0.6 (±0.6)	0.4 (±0.5)	<0.001
Tea^b^	1 (±1.2)	1.1 (±1.4)	1.2 (±1.7)	1.3 (±1.6)	<0.001
Coffee^b^	3.4 (±2.4)	3.4 (±2.2)	3.5 (±2.1)	3.4 (±2)	<0.001
Soft drinks^c^	1.3 (±1.7)	1.1 (±1.5)	1 (±1.4)	0.6 (±1.3)	<0.001
Food consumption per week	​	​	​	​	​
Low-fat cheese^b^	0.4 (±1.2)	0.7 (±1.5)	0.9 (±1.8)	1.2 (±1.8)	<0.001
Offal^c^	0.3 (±0.7)	0.3 (±0.7)	0.2 (±0.6)	0.2 (±0.5)	<0.001
Use of olive/canola oil^b^	0.1 (±0.3)	0.1 (±0.3)	0.2 (±0.4)	0.3 (±0.5)	<0.001
Chips^c^	1.7 (±1.8)	1.5 (±1.7)	1.4 (±1.7)	1.2 (±1.6)	<0.001
Chocolate^b^	0.8 (±1.3)	1 (±1.5)	1.1 (±1.5)	1.3 (±1.7)	<0.001
Nuts^b^	0.3 (±0.5)	0.3 (±0.7)	0.4 (±0.8)	0.5 (±1.2)	<0.001
Wine^b^	1.2 (±1.7)	1.5 (±1.8)	1.7 (±1.8)	2.2 (±2)	<0.001
Beer^b^	3.8 (±6.8)	3.9 (±6)	4 (±5.6)	4.2 (±5.1)	<0.001

a Civil status was not available for the women cohort.

b Anti-inflammatory.

c Pro-inflammatory.

**Table 2.** Associations between AIDI and bladder cancer risk by stage (1998–2020).

AIDI score	Case	Person-year	Baseline exposure–1998			Repeated measures of AIDI (1998 and 2009)^a^		
			HRs (95% CIs)			HRs (95% CIs)		
			Model 1^b^	Model 2^c^	Model 3^d^	Model 1^b^	Model 2^c^	Model 3^d^
Bladder cancer	1,165	​	​	​	​	​	​	​
Q1 (0–4)	411	442,516	Reference	Reference	Reference	Reference	Reference	Reference
Q2 (5)	262	294,163	0.97 (0.83–1.13)	1 (0.85–1.16)	1 (0.85–1.16)	0.95 (0.80–1.11)	0.97 (0.82–1.14)	0.97 (0.82–1.14)
Q3 (6–7)	218	489,874	0.86 (0.73–1.01)	0.89 (0.76–1.06)	0.90 (0.76–1.06)	0.86 (0.74–1.01)	0.91 (0.78–1.07)	0.91 (0.78–1.07)
Q4 (8–13)	274	206,649	0.81 (0.70–0.95)	0.87 (0.74–1.02)	0.87 (0.74–1.02)	0.67 (0.56–0.81)	0.74 (0.62–0.89)	0.74 (0.61–0.89)
*P* value for trend	​	​	0.03	0.22	0.23	<0.001	0.01	0.01
NMIBC^e^^,^^f^	249	​	​	​	​	​	​	​
Q1 (0–4)	91	438,970	Reference	Reference	Reference	Reference	Reference	Reference
Q2 (5)	61	292,162	1.02 (0.74–1.42)	1.07 (0.77–1.49)	1.07 (0.77–1.49)	0.96 (0.65–1.41)	1.00 (0.67–1.47)	1 (0.67–1.47)
Q3 (6–7)	65	486,821	0.65 (0.44–0.96)	0.69 (0.47–1.02)	0.69 (0.47–1.02)	0.95 (0.67–1.35)	1.04 (0.73–1.49)	1.04 (0.73–1.48)
Q4 (8–13)	32	205,532	0.81 (0.59–1.13)	0.92 (0.66–1.29)	0.92 (0.66–1.29)	0.72 (0.49–1.07)	0.86 (0.58–1.29)	0.86 (0.57–1.28)
*P* value for trend	​	​	0.10	0.19	0.19	0.31	0.74	0.73
MIBC^f^^,^^g^	201	​	​	​	​	​	​	​
Q1 (0–4)	72	438,532	Reference	Reference	Reference	Reference	Reference	Reference
Q2 (5)	50	291,827	1.03 (0.72–1.47)	1.05 (0.73–1.51)	1.05 (0.73–1.51)	0.83 (0.57–1.21)	0.85 (0.58–1.24)	0.85 (0.58–1.24)
Q3 (6–7)	61	486,623	0.97 (0.67–1.41)	1 (0.69–1.46)	1 (0.69–1.46)	0.68 (0.47–0.98)	0.71 (0.49–1.02)	0.71 (0.49–1.02)
Q4 (8–13)	18	205,304	0.55 (0.36–0.82)	0.57 (0.38–0.86)	0.57 (0.38–0.86)	0.34 (0.21–0.54)	0.35 (0.22–0.57)	0.35 (0.22–0.57)
*P* value for trend	​	​	0.02	0.03	0.03	0.03	<0.001	<0.001

AIDI score: Cutpoints for the AIDI categories were defined using the distribution in the overall study population (population-wide).

a Cumulative-average method.

b Model 1 adjusted for age and sex.

c Model 2 additionally adjusted for smoking (pack-years), BMI, education, employment status, and average caloric intake which was centered at the mean by sex.

d Model 3 additionally adjusted for diabetes, hypertension, and family history of cancer.

e NMIBC (Tis or Ta or T1 and N0 and M0).

f For cancer cases diagnosed after 2004.

g MIBC (≥T2 or ≥N1 or ≥M1).

Tables 3 and 4 present the associations between the AIDI and bladder cancer risk by stage, additionally adjusted for different dietary components. Table 3, adjusted for anti-inflammatory components of AIDI (thus evaluating the effect of pro-inflammatory components of AIDI), shows that for total bladder cancer, the highest AIDI quartile (Q4, score 5–10) at baseline was associated with a reduced risk (HR = 0.80; 95% CI, 0.65–0.98), though this association was not significant in the repeated measures. No significant associations were observed for NMIBC in either analysis. However, there was a trend toward a reduced risk for MIBC in the highest quartile at baseline (HR = 0.60; 95% CI, 0.36–1.02), which became less pronounced in the repeated-measures analysis. Table 4, which is additionally adjusted for pro-inflammatory components (thus evaluating anti-inflammatory components), did not show significant associations with total bladder cancer, NMIBC, or MIBC in either the baseline or repeated measures. Supplementary Table S4 provides the baseline characteristics of Swedish men and women by quartiles of the AIDI (maximum score = 13).

**Table 3.** Associations between only anti-inflammatory components of AIDI and bladder cancer risk, overall and by disease stage (1998–2020).

AIDI score	Case	Person-year	Baseline exposure–1998	Repeated measures of AIDI (1998 and 2009)^a^
			HRs (95% CIs)	HRs (95% CIs)
			Model 3^b^	Model 3^b^
Bladder cancer	1,165	​	​	​
Q1 (0–2)	411	333,328	Reference	Reference
Q2 (3)	262	387,610	0.97 (0.82–1.14)	1.02 (0.87–1.19)
Q3 (4)	218	349,995	0.85 (0.71–1.02)	0.92 (0.78–1.10)
Q4 (5–10)	274	362,268	0.80 (0.65–0.98)	0.92 (0.77–1.09)
*P* value for trend	​	​	0.12	0.58
NMIBC^c^^,^^d^	249	​	​	​
Q1 (0–2)	91	331,036	Reference	Reference
Q2 (3)	61	385,075	1.08 (0.77–1.53)	1.07 (0.78–1.48)
Q3 (4)	65	347,339	0.70 (0.46–1.07)	0.69 (0.46–1.02)
Q4 (5–10)	32	360,034	0.95 (0.61–1.48)	0.91 (0.63–1.31)
*P* value for trend	​	​	0.23	0.19
MIBC^d^^,^^e^	201	​	​	​
Q1 (0–2)	72	330,849	Reference	Reference
Q2 (3)	50	384,653	1.07 (0.73–1.57)	1.14 (0.79–1.64)
Q3 (4)	61	346,994	1.04 (0.68–1.57)	1.15 (0.78–1.69)
Q4 (5–10)	18	359,790	0.60 (0.36–1.02)	0.72 (0.46–1.13)
*P* value for trend	​	​	0.09	0.17

a Cumulative-average method.

b Model 3 adjusted for age, sex, smoking (pack-years), BMI, education, employment status, average caloric intake which was centered at the mean by sex, diabetes, hypertension, and family history of cancer and anti-inflammatory components of AIDI score.

c NMIBC (Tis or Ta or T1 and N0 and M0).

d For cancer cases diagnosed after 2004.

e MIBC (≥T2 or ≥N1 or ≥M1).

**Table 4.** Associations between only pro-inflammatory components of AIDI and bladder cancer risk, overall and by disease stage (1998–2020).

AIDI score	Case	Person-year	Baseline exposure–1998	Repeated measures of AIDI (1998 and 2009)^d^
			HRs (95% CIs)	HRs (95% CIs)
			Model 3^a^	Model 3^a^
Bladder cancer	1,165	​	​	​
Q1 (0–1)	411	404,836	Reference	Reference
Q2 (2)	262	479,307	1.02 (0.87–1.20)	0.98 (0.83–1.17)
Q3 (3)	218	396,985	0.94 (0.79–1.12)	1.08 (0.93–1.25)
Q4 (4–5)	274	152,074	0.94 (0.78–1.13)	1.03 (0.85–1.25)
*P* value for trend	​	​	0.74	0.69
NMIBC^be^	249	​	​	​
Q1 (0–1)	91	401,451	Reference	Reference
Q2 (2)	61	476,069	1.08 (0.77–1.51)	1.16 (0.81–1.67)
Q3 (3)	65	394,691	0.70 (0.47–1.06)	1 (0.71–1.39)
Q4 (4–5)	32	151,274	0.94 (0.64–1.40)	1.15 (0.77–1.71)
*P* value for trend	​	​	0.23	0.75
MIBC^ce^	201	​	​	​
Q1 (0–1)	72	401,134	Reference	Reference
Q2 (2)	50	475,527	1.08 (0.74–1.56)	0.97 (0.65–1.45)
Q3 (3)	61	394,474	1.05 (0.70–1.56)	1.15 (0.81–1.62)
Q4 (4–5)	18	151,151	0.61 (0.38–0.98)	0.68 (0.40–1.14)
*P* value for trend	​	​	0.07	0.20

a Model 3 adjusted for age, sex, smoking (pack-years), BMI, education, employment status, average caloric intake which was centered at the mean by sex, diabetes, hypertension, and family history of cancer and pro-inflammatory components of AIDI score.

b NMIBC (Tis or Ta or T1 and N0 and M0).

c MIBC (≥T2 or ≥N1 or ≥M1).

d Cumulative-average method.

e For cancer cases diagnosed after 2004.

Sample reduction when adding alcohol and/or physical activity (complete-case analysis) is shown in Supplementary Table S5, and sensitivity analyses with additional adjustment for physical activity and alcohol did not materially change risk estimates (Supplementary Table S6). Lag analyses excluding bladder cancer cases diagnosed within the first 2 and 3 years after the start of follow-up (fully adjusted model 3) for both baseline (1998) and repeated-measures (cumulative-average) exposures are shown in Supplementary Table S7. In addition, stratified analyses by baseline smoking status (never vs. ever; fully adjusted model 3) showed broadly similar associations across strata, and formal tests for interaction did not indicate effect modification (Supplementary Table S8).

## Discussion

In this large cohort with 22 years of follow-up, higher AIDI scores were associated with lower bladder cancer risk, especially for muscle-invasive disease, suggesting that an anti-inflammatory dietary pattern may be linked to reduced bladder cancer risk. The models were adjusted for age, sex, smoking, BMI, education, employment status, average caloric intake, diabetes, hypertension, and family history of cancer, and associations were stronger when using the cumulative-average AIDI based on repeated FFQs compared with baseline AIDI. No significant differences were observed between sexes, and additional models adjusting separately for anti- and pro-inflammatory components showed weak or no associations, especially in the repeated-measures analysis.

Studies have investigated the association between dietary pro-inflammatory potential and bladder cancer risk. A large Italian case–control study found that individuals with higher dietary inflammatory index (DII) scores had an increased risk of bladder cancer, with stronger associations observed among females, older adults, and never smokers (24). Similarly, an Iranian case–control study reported higher bladder cancer risk for subjects with higher DII scores, particularly among current/ex-smokers (25). A more recent case–control study from the United States, National Health and Nutrition Examination Survey, also demonstrated a positive association between pro-inflammatory diets and bladder cancer risk (17). However, a meta-analysis reported nonsignificant associations between DII and bladder cancer risk in both case–control and cohort studies (26, 27). In our study, no clear associations were observed when adjusting separately for anti- and pro-inflammatory components of the diet. The inconsistency in the literature, along with our findings, suggests that the protective effects may not be attributable to individual dietary components alone. Rather, they may be driven by the combined effect of a dietary pattern characterized by both increased intake of anti-inflammatory foods and reduced intake of pro-inflammatory foods.

Fruits and vegetables were included as components of the AIDI score and were comprehensively assessed through the FFQ. Research on the relationship between fruit and vegetable consumption and bladder cancer risk has yielded mixed results. Some studies suggest that higher intake of fruits and vegetables may reduce bladder cancer risk (26, 27). Specifically, pomegranates, cranberries, and citrus fruits have shown potential protective effects (27). A pooled analysis of case–control studies found that higher consumption of total fruits, citrus fruits, pome fruits, tropical fruits, and total vegetables was associated with decreased bladder cancer risk (26). Additionally, a meta-analysis of 27 studies reported that higher consumption of both fruits and vegetables was associated with a reduced risk of bladder cancer (28). However, others have found no significant association between fruit and vegetable intake and bladder cancer risk (29, 30). A meta-analysis of prospective studies reported no consistent evidence for a protective role of fruits and vegetables in bladder cancer prevention (29). The conflicting findings highlight the need for further research to clarify the relationship between fruit and vegetable consumption and bladder cancer risk.

Although an anti-inflammatory diet was inversely associated with bladder cancer risk overall, the association was particularly pronounced for MIBC. Compared with NMIBC, MIBC is characterized by a more inflamed tumor environment, with higher expression of pro-inflammatory cytokines and activation of inflammation-related signaling pathways (31, 32). This heightened inflammatory state may make MIBC more susceptible to the systemic effects of diet-induced inflammation. Anti-inflammatory dietary patterns, which are rich in fiber, antioxidants, and unsaturated fats, lower CRP, IL6, and TNFα, potentially reducing inflammation in the tumor microenvironment (6, 33, 34). These systemic changes may, in turn, influence the tumor microenvironment, reducing protumorigenic signaling and slowing disease progression (35, 36). Therefore, in the context of MIBC, in which inflammation plays a more central role in tumor aggressiveness and progression, the protective effect of an anti-inflammatory diet may be more pronounced.

Diet-induced systemic inflammation may be more relevant to processes involved in invasive transformation, such as tissue remodeling, immune evasion, and other microenvironment-dependent programs (37), than to those involved in the development of many noninvasive papillary tumors. Moreover, NMIBC encompasses a heterogeneous spectrum of lesions with differing progression potential [e.g., low-risk papillary tumors vs. high-risk T1 (lamina propria–invasive) tumors or carcinoma *in situ* (38)], and analyzing NMIBC as a single group may attenuate associations if the diet–inflammation axis primarily relates to the subset with aggressive potential. Additionally, MIBC is likely less susceptible to detection bias compared with NMIBC (37, 38). Individuals with low AIDI may have poorer overall health and may thus be subjected to more medical examinations, rendering more chance findings of bladder cancer. Our finding of a stronger association between AIDI and MIBC may alleviate this concern.

A major strength of our study is its large, population-based prospective design, with a follow-up period extending up to 22 years. Dietary intake was comprehensively assessed at two distinct time points, 12 years apart, allowing us to capture both baseline and long-term dietary patterns. Bladder cancer cases were identified through the highly reliable Swedish Cancer Registry, and individuals with a history of cancer (except nonmelanoma skin cancer) were systematically excluded to minimize potential bias. Our analyses were adjusted for possible confounders, including key established risk factors for bladder cancer: smoking, BMI, hypertension, and diabetes. In addition, we incorporated detailed sociodemographic data, migration history, and mortality status, retrieved from high-quality national registers, which further strengthened the validity and robustness of our results. The free healthcare access in Sweden likely reduces differential access to diagnostics and treatment, thereby minimizing bias related to healthcare utilization.

There are several limitations of this study that should be acknowledged. Although dietary intake was assessed using a validated FFQ, self-reported data are inherently subject to recall bias and measurement error (38). Additional limitations include residual confounding and possible overadjustment when separating AIDI components. Although such misclassification may lead to underestimation or overestimation of associations, it is likely to be nondifferential with respect to bladder cancer outcomes, thereby biasing results toward the null and yielding conservative and comparable estimates. Furthermore, most participants completed the FFQ within a few months, which reduces concerns related to seasonal variability in dietary reporting. Although we collected dietary data at two time points, spaced 12 years apart, it is possible that we did not fully capture long-term dietary patterns or changes in diet over time. Although our analyses adjusted for a broad set of established confounders, including smoking, BMI, hypertension, and diabetes, residual confounding cannot be excluded, particularly with respect to physical activity, occupational exposures, and nonsteroidal anti-inflammatory drug (NSAID) use, which were not accounted for in the current models. In addition, analyses that adjusted separately for anti- and pro-inflammatory components may have introduced overadjustment bias, as these variables form the core components of the AIDI score. Controlling for them individually likely reduced variance and may have attenuated the observed associations because of multicollinearity and conceptual overlap. Although our models adjusted for energy intake (centered at the sex-specific mean), this pattern may reflect broader differences in overall dietary habits, health consciousness, or dietary restraint that are not fully captured by measured covariates. Therefore, some residual confounding by overall diet quality or caloric restriction cannot be excluded and should be considered when interpreting the magnitude of the observed associations. Additionally, identifying which specific foods drive the association would be informative, but the present study focused on the AIDI as a predefined composite measure of overall dietary inflammatory potential. Detailed component-specific analyses were beyond the scope of this work and warrant future investigation. Lastly, because the cohort is based in Sweden, generalizability to more ethnically or socioeconomically diverse populations may be limited.

In conclusion, in this large, prospective Swedish cohort with up to 22 years of follow-up, higher anti-inflammatory diet scores were associated with lower bladder cancer risk, particularly for muscle-invasive disease. These findings support the potential role of diet-induced inflammation in bladder cancer development and progression and highlight the importance of considering overall dietary patterns rather than individual components in cancer prevention strategies.

## Supplementary Material

Supplementary Table 2 Supplementary Table 2 reports hazard ratios (HRs) and 95% confidence intervals for bladder cancer risk across quartiles of the Anti-Inflammatory Diet Index (AIDI) among Swedish women (1998–2020), overall and stratified by tumour stage (non-muscle invasive and muscle invasive). Results are shown for baseline AIDI (1998) and for AIDI modelled as a repeated measure (1998 and 2009; cumulative-average method), with tests for linear trend. Estimates are presented from three progressively adjusted Cox models: Model 1 (age and sex), Model 2 (additionally smoking pack-years, BMI, education, employment status, and mean-centred energy intake), and Model 3 (additionally diabetes, hypertension, and family history of cancer). Case counts and person-years are provided for each AIDI category, and stage-specific analyses include cases diagnosed from 2004 onwards.

Supplementary Table 3 Supplementary Table 3 reports hazard ratios (HRs) and 95% confidence intervals for bladder cancer risk across quartiles of the Anti-Inflammatory Diet Index (AIDI) among Swedish men (1998–2020), overall and stratified by tumour stage (non-muscle invasive and muscle invasive). Results are shown for baseline AIDI (1998) and for AIDI modelled as a repeated measure (1998 and 2009; cumulative-average method), with tests for linear trend. Estimates are presented from three progressively adjusted Cox models: Model 1 (age and sex), Model 2 (additionally smoking pack-years, BMI, education, employment status, and mean-centred energy intake), and Model 3 (additionally diabetes, hypertension, and family history of cancer). Case counts and person-years are provided for each AIDI category, and stage-specific analyses include cases diagnosed from 2004 onwards.

Supplementary Table 4 Supplementary Table 4 summarises baseline characteristics of Swedish men and women by quartiles of the Anti-Inflammatory Diet Index (AIDI; maximum score 13), including participant numbers, follow-up time (person-years), age, education, smoking pack-years, BMI, employment status, history of hypertension and diabetes, family history of cancer, and total energy intake. It also presents mean (±SD) consumption of the AIDI food components (anti-inflammatory and pro-inflammatory items) by quartile, together with p-values for overall differences across quartiles; civil status is reported for men only because it was not available in the women’s cohort.

Supplementary Table 5 Supplementary Table 5 quantifies the reduction in analytic sample size when restricting to complete cases for alcohol intake and/or physical activity, reporting the remaining number of participants, bladder cancer cases, and person-years, as well as the numbers and percentages excluded due to missing data. Estimates are shown for the overall bladder cancer dataset and for the non-muscle invasive (NMIBC) and muscle invasive (MIBC) stage-specific datasets.

Supplementary Table 6 Supplementary Table 6 presents sensitivity analyses examining whether additional adjustment for alcohol intake and/or leisure-time physical activity influences the association between the Anti-Inflammatory Diet Index (AIDI) and bladder cancer risk. Hazard ratios (HRs) and 95% confidence intervals are shown for quartiles of AIDI (Q2–Q4 vs Q1) and p-values for trend, using Model 3 for baseline AIDI (1998) and for AIDI modelled as a repeated measure (1998 and 2009; cumulative-average method). Results are reported for the full sample and for complete-case subsets for alcohol, physical activity, and both combined, with models additionally adjusted for total alcohol intake (g/day) and/or leisure-time exercise (hours/week category) as indicated.

Supplementary Table 7 Supplementary Table 7 shows lagged sensitivity analyses evaluating the association between the Anti-Inflammatory Diet Index (AIDI) and bladder cancer risk after excluding cases diagnosed early in follow-up (no lag, 2-year lag, and 3-year lag). For each lag period, the table reports the number of excluded cases, remaining cases and person-years, hazard ratios (HRs) with 95% confidence intervals for AIDI quartiles (Q2–Q4 vs Q1), and p-values for trend, using fully adjusted Model 3 for baseline AIDI (1998) and for AIDI modelled as a repeated measure (1998 and 2009; cumulative-average method).

Supplementary Table 8 Supplementary Table 8 reports stratified associations between the Anti-Inflammatory Diet Index (AIDI) and bladder cancer risk by baseline smoking status (never vs ever smokers). Hazard ratios (HRs) and 95% confidence intervals are presented for AIDI quartiles (Q2–Q4 vs Q1) for baseline AIDI (1998) and for AIDI modelled as a repeated measure (1998 and 2009; cumulative-average method), including p-values for trend within each smoking stratum. Fully adjusted Model 3 covariates are specified, and p-values for interaction between AIDI trend and smoking group are provided for both exposure specifications.

Supplementary Table 1 Supplementary Table 1 lists the food frequency questionnaire (FFQ)-derived components of the Anti-Inflammatory Diet Index (AIDI), indicates whether each component is classified as anti-inflammatory or pro-inflammatory, specifies the intake cut-offs used to assign a score of 1, and defines the binary scoring rule (1 if the criterion is met; otherwise 0).

## Data Availability

This study used data from the Swedish Medical Information Platform for Epidemiological Research (SIMPLER). Due to legal and ethical restrictions, access to SIMPLER data is limited. The dataset is not publicly available but can be obtained upon reasonable request and approval from the relevant data custodians. Researchers interested in accessing SIMPLER data should contact simpler@surgsci.uu.se and follow the official application procedures. Further information on data access is available at https://www.simpler4health.se.
