# Supplementary material for: Anti-inflammatory Diet Index and Bladder Cancer Risk by Stage: A 22-Year Prospective Swedish Cohort Study (1998–2020)
Source: Cancer Epidemiol Biomarkers Prev. 2026 Mar 31;35(6):1019–26. doi: 10.1158/1055-9965.EPI-25-1733 (PMC13227089; doi:10.1158/1055-9965.EPI-25-1733)
Supplement: Supplementary Table 2 — reports hazard ratios (HRs) and 95% confidence intervals for bladder cancer risk across quartiles of the Anti-Inflammatory Diet Index (AIDI) among Swedish women (1998–2020), overall and stratified by tumour stage (non-muscle invasive and muscle invasive). Results are shown for baseline AIDI (1998) and for AIDI modelled as a repeated measure (1998 and 2009; cumulative-average method), with tests for linear trend. Estimates are presented from three progressively adjusted Cox models: Model 1 (age and sex), Model 2 (additionally smoking pack-years, BMI, education, employment status, and mean-centred energy intake), and Model 3 (additionally diabetes, hypertension, and family history of cancer). Case counts and person-years are provided for each AIDI category, and stage-specific analyses include cases diagnosed from 2004 onwards. [file epi-25-1733_supplementary_table_2_suppst2.docx]

**Supplementary Table 2.** Associations between Anti-Inflammatory Diet Index (AIDI) and Bladder Cancer Risk by Stage among Swedish Women (1998-2020)

|  |  |  | Baseline exposure-1998 | | |  | Repeated measure of AIDI (1998 & 2009)^¶^ | | |
| --- | --- | --- | --- | --- | --- | --- | --- | --- | --- |
| AIDI score |  |  | Hazard ratios (95% confidence intervals) | | |  | Hazard ratios (95% confidence intervals) | | |
|  | Cases | Person-years | Model 1^a^ | Model 2 ^b^ | Model 3 ^c^ |  | Model 1^a^ | Model 2 ^b^ | Model 3 ^c^ |
| Bladder cancer | 213 |  |  |  |  |  |  |  |  |
| Q1 (0-4) | 62 | 183311 | Reference | Reference | Reference |  | Reference | Reference | Reference |
| Q2 (5) | 41 | 135619 | 0.89 (0.60, 1.32) | 0.90 (0.61, 1.34) | 0.90 (0.61, 1.34) |  | 1.11 (0.77, 1.61) | 1.13 (0.78, 1.65) | 1.13 (0.78, 1.65) |
| Q3 (6-7) | 71 | 250236 | 0.83 (0.56, 1.23) | 0.85 (0.57, 1.27) | 0.85 (0.57, 1.28) |  | 0.78 (0.53, 1.15) | 0.81 (0.54, 1.20) | 0.80 (0.54, 1.20) |
| Q4 (8-13) | 39 | 123855 | 0.90 (0.64, 1.26) | 0.93 (0.66, 1.33) | 0.93 (0.66, 1.32) |  | 0.60 (0.39, 0.91) | 0.63 (0.41, 0.97) | 0.63 (0.40, 0.97) |
| P value for trend |  |  | 0.81 | 0.88 | 0.89 |  | 0.01 | 0.03 | 0.03 |
| Non-Muscle Invasive BC *^d *^* | 48 |  |  |  |  |  |  |  |  |
| Q1 (0-4) | 14 | 182710 | Reference | Reference | Reference |  | Reference | Reference | Reference |
| Q2 (5) | 11 | 135300 | 1.04 (0.47, 2.28) | 1.10 (0.50, 2.44) | 1.11 (0.50, 2.45) |  | 1.14 (0.47, 2.79) | 1.28 (0.52, 3.17) | 1.27 (0.51, 3.15) |
| Q3 (6-7) | 13 | 249631 | 0.82 (0.36, 1.90) | 0.91 (0.39, 2.13) | 0.91 (0.39, 2.12) |  | 0.89 (0.37, 2.12) | 1.02 (0.42, 2.47) | 1.00 (0.41, 2.43) |
| Q4 (8-13) | 10 | 123521 | 0.76 (0.36, 1.60) | 0.91(0.43, 1.95) | 0.91(0.42, 1.94) |  | 0.87 (0.36, 2.07) | 1.15 (0.47, 2.85) | 1.14 (0.46, 2.81) |
| P value for trend |  |  | 0.84 | 0.97 | 0.96 |  | 0.89 | 0.93 | 0.93 |
| Muscle Invasive BC*^e *^* | 47 |  |  |  |  |  |  |  |  |
| Q1 (0-4) | 15 | 182716 | Reference | Reference | Reference |  | Reference | Reference | Reference |
| Q2 (5) | 10 | 135239 | 0.91 (0.41, 2.02) | 0.95 (0.43, 2.13) | 0.95 (0.43, 2.13) |  | 0.88 (0.44, 1.78) | 0.92 (0.45, 1.86) | 0.92 (0.45, 1.86) |
| Q3 (6-7) | 14 | 249598 | 0.77(0.34, 1.77) | 0.82 (0.36, 1.90) | 0.82 (0.36, 1.90) |  | 0.32 (0.14, 0.78) | 0.34 (0.14, 0.82) | 0.34 (0.14, 0.82) |
| Q4 (8-13) | 8 | 123492 | 0.68(0.33, 1.44) | 0.74 (0.34, 1.58) | 0.73 (0.34, 1.57) |  | 0.29 (0.11, 0.71) | 0.29 (0.11, 0.77) | 0.29 (0.11, 0.77) |
| P value for trend |  |  | 0.77 | 0.87 | 0.86 |  | 0.01 | 0.01 | 0.01 |

^a^ Model1 adjusted for age and sex
^b^ Model2 additionally adjusted for smoking (pack years), BMI, education, employment status, and average caloric intake which was centered at the mean by sex
^c^ Model3 additionally adjusted for diabetes, hypertension and family history of cancer
^d^ Non-muscle Invasive Bladder cancer (Tis or Ta or T1and N0 and M0)
^e^ Muscle Invasive BC (>=T2 or >=N1 or >=M1)
^¶^ cumulative-average method
* For cancer cases diagnosed after 2004
