# Supplementary material for: Anti-inflammatory Diet Index and Bladder Cancer Risk by Stage: A 22-Year Prospective Swedish Cohort Study (1998–2020)
Source: Cancer Epidemiol Biomarkers Prev. 2026 Mar 31;35(6):1019–26. doi: 10.1158/1055-9965.EPI-25-1733 (PMC13227089; doi:10.1158/1055-9965.EPI-25-1733)
Supplement: Supplementary Table 3 — reports hazard ratios (HRs) and 95% confidence intervals for bladder cancer risk across quartiles of the Anti-Inflammatory Diet Index (AIDI) among Swedish men (1998–2020), overall and stratified by tumour stage (non-muscle invasive and muscle invasive). Results are shown for baseline AIDI (1998) and for AIDI modelled as a repeated measure (1998 and 2009; cumulative-average method), with tests for linear trend. Estimates are presented from three progressively adjusted Cox models: Model 1 (age and sex), Model 2 (additionally smoking pack-years, BMI, education, employment status, and mean-centred energy intake), and Model 3 (additionally diabetes, hypertension, and family history of cancer). Case counts and person-years are provided for each AIDI category, and stage-specific analyses include cases diagnosed from 2004 onwards. [file epi-25-1733_supplementary_table_3_suppst3.docx]

**Supplementary Table 3.** Associations between Anti-Inflammatory Diet Index (AIDI) and Bladder Cancer Risk by Stage among Swedish Men (1998-2020)

|  |  |  | Baseline exposure-1998 | | |  | Repeated measure of AIDI (1998 & 2009)^¶^ | | |
| --- | --- | --- | --- | --- | --- | --- | --- | --- | --- |
| AIDI score |  |  | Hazard ratios (95% confidence intervals) | | |  | Hazard ratios (95% confidence intervals) | | |
|  | Cases | Person-years | Model 1^a^ | Model 2 ^b^ | Model 3 ^c^ |  | Model 1^a^ | Model 2 ^b^ | Model 3 ^c^ |
| Bladder cancer | 952 |  |  |  |  |  |  |  |  |
| Q1 (0-4) | 349 | 259205 | Reference | Reference | Reference |  | Reference | Reference | Reference |
| Q2 (5) | 221 | 158544 | 0.98 (0.83, 1.17) | 1.01 (0.86, 1.20) | 1.01 (0.86, 1.20) |  | 0.90 (0.75, 1.08) | 0.93 (0.78, 1.12) | 0.93 (0.78, 1.12) |
| Q3 (6-7) | 287 | 239639 | 0.87 (0.72, 1.04) | 0.90 (0.75, 1.08) | 0.90 (0.75, 1.09) |  | 0.88 (0.74, 1.04) | 0.94 (0.78, 1.12) | 0.93 (0.78, 1.11) |
| Q4 (8-12) | 95 | 82794 | 0.79 (0.66, 0.93) | 0.85 (0.71, 1.01) | 0.85 (0.71, 1.01) |  | 0.69 (0.56, 0.84) | 0.77 (0.63, 0.94) | 0.76 (0.62, 0.94) |
| P value for trend |  |  | 0.03 | 0.18 | 0.19 |  | 0.00 | 0.06 | 0.07 |
| Non-Muscle Invasive BC *^d *^* | 201 |  |  |  |  |  |  |  |  |
| Q1 (0-4) | 77 | 256260 | Reference | Reference | Reference |  | Reference | Reference | Reference |
| Q2 (5) | 50 | 156862 | 1.02 (0.71, 1.46) | 1.07 (0.75, 1.53) | 1.07 (0.75, 1.53) |  | 0.91 (0.59, 1.40) | 0.93 (0.61, 1.44) | 0.94 (0.61, 1.44) |
| Q3 (6-7) | 52 | 237190 | 0.61 (0.39, 0.94) | 0.64 (0.41, 0.99) | 0.64 (0.41, 0.99) |  | 0.95 (0.65, 1.40) | 1.03 (0.70, 1.52) | 1.03 (0.69, 1.51) |
| Q4 (8-12) | 22 | 82011 | 0.83 (0.58, 1.19) | 0.93 (0.64, 1.35) | 0.93 (0.64, 1.35) |  | 0.68 (0.43, 1.05) | 0.79 (0.50, 1.24) | 0.79 (0.50, 1.23) |
| P value for trend |  |  | 0.11 | 0.16 | 0.17 |  | 0.26 | 0.58 | 0.58 |
| Muscle Invasive BC *^e*^* | 154 |  |  |  |  |  |  |  |  |
| Q1 (0-4) | 57 | 255816 | Reference | Reference | Reference |  | Reference | Reference | Reference |
| Q2 (5) | 40 | 156588 | 1.06 (0.71, 1.60) | 1.09 (0.72, 1.63) | 1.08 (0.72, 1.63) |  | 0.80 (0.51, 1.24) | 0.82 (0.52, 1.29) | 0.82 (0.52, 1.29) |
| Q3 (6-7) | 47 | 237025 | 1.05 (0.69, 1.59) | 1.08 (0.71, 1.65) | 1.08 (0.71, 1.65) |  | 0.80 (0.53, 1.21) | 0.84 (0.55, 1.27) | 0.84 (0.55, 1.27) |
| Q4 (8-12) | 10 | 81812 | 0.49 (0.29, 0.80) | 0.51 (0.31, 0.85) | 0.51(0.31, 0.85) |  | 0.35 (0.20, 0.61) | 0.37 (0.21, 0.66) | 0.38 (0.21, 0.66) |
| P value for trend |  |  | 0.02 | 0.03 | 0.03 |  | 0.02 | 0.01 | 0.01 |

^a^ Model1 adjusted for age and sex
^b^ Model2 additionally adjusted for smoking (pack years), BMI, education, employment status, and average caloric intake which was centered at the mean by sex
^c^ Model3 additionally adjusted for diabetes, hypertension and family history of cancer
^d^ Non-muscle Invasive Bladder cancer (Tis or Ta or T1and N0 and M0)
^e^ Muscle Invasive BC (>=T2 or >=N1 or >=M1)
^¶^ cumulative-average method
* For cancer cases diagnosed after 2004
