# Supplementary material for: Anti-inflammatory Diet Index and Bladder Cancer Risk by Stage: A 22-Year Prospective Swedish Cohort Study (1998–2020)
Source: Cancer Epidemiol Biomarkers Prev. 2026 Mar 31;35(6):1019–26. doi: 10.1158/1055-9965.EPI-25-1733 (PMC13227089; doi:10.1158/1055-9965.EPI-25-1733)
Supplement: Supplementary Table 4 — summarises baseline characteristics of Swedish men and women by quartiles of the Anti-Inflammatory Diet Index (AIDI; maximum score 13), including participant numbers, follow-up time (person-years), age, education, smoking pack-years, BMI, employment status, history of hypertension and diabetes, family history of cancer, and total energy intake. It also presents mean (±SD) consumption of the AIDI food components (anti-inflammatory and pro-inflammatory items) by quartile, together with p-values for overall differences across quartiles; civil status is reported for men only because it was not available in the women’s cohort. [file epi-25-1733_supplementary_table_4_suppst4.docx]

**Supplement Table 4**. Descriptive baseline characteristics of Swedish men and women by quartiles of the anti-inflammatory diet index (AIDI; maximum score=13)

|  | Male | | | |  | Female | | | |  |
| --- | --- | --- | --- | --- | --- | --- | --- | --- | --- | --- |
|  | Quartiles of AIDI | | | |  | Quartiles of AIDI | | | |  |
| Characteristics | 0-4 | 5 | 06 | 7 -13 | p.over all | 0-4 | 5 | 06 | 7 -13 | p.over all |
| Number of participants | 15246 | 9302 | 8319 | 10324 |  | 9777 | 7144 | 7379 | 11801 |  |
| Pyears | 17.0 (±7.1) | 17.0 (±7.1) | 17.1 (±7.1) | 17.4 (±6.9) | <0.001 | 18.7 (±6.1) | 19.0 (±6.0) | 19.1 (±5.9) | 19.8 (±5.5) | <0.001 |
| Age at baseline ±SD, years | 60.6 (±9.6) | 61.2 (±9.7) | 61.5 (±9.8) | 61.4 (±9.7) | <0.001 | 63.1 (±9.4) | 63.0 (±9.3) | 63.2 (±9.3) | 62.1 (±9.0) | <0.001 |
| Education |  |  |  |  | <0.001 |  |  |  |  | <0.001 |
| 1. Primary | 5906 (38.7%) | 3348 (36.0%) | 2789 (33.5%) | 2688 (26.0%) |  | 4837 (49.5%) | 3325 (46.5%) | 3119 (42.3%) | 3803 (32.2%) |  |
| 1. Secondary | 7532 (49.4%) | 4598 (49.4%) | 4070 (48.9%) | 5060 (49.0%) |  | 3831 (39.2%) | 2820 (39.5%) | 2911 (39.4%) | 4764 (40.4%) |  |
| 1. University | 1808 (11.9%) | 1356 (14.6%) | 1460 (17.6%) | 2576 (25.0%) |  | 1109 (11.3%) | 999 (14.0%) | 1349 (18.3%) | 3234 (27.4%) |  |
| Smoking Pack-years | 13.6 (±16.3) | 12.5 (±15.7) | 11.4 (±15.0) | 10.2 (±14.2) | <0.001 | 7.0 (±11.2) | 6.9 (±11.0) | 6.30 (±10.3) | 6.05 (±9.8) | <0.001 |
| BMI | 26.1 (±3.6) | 25.8 (±3.4) | 25.7 (±3.3) | 25.3 (±3.0) | <0.001 | 25.6 (±4.3) | 25.2 (±4.0) | 24.9 (±3.8) | 24.6 (±3.6) | <0.001 |
| Civil status ^c^ |  |  |  |  | <0.001 |  |  |  |  | --- |
| 1. Single | 1148 (7.6%) | 608 (6.6%) | 535 (6.5%) | 535 (5.2%) |  | --- | --- | --- | --- |  |
| 1. Married/ cohabiting | 12321 (81.1%) | 7670 (82.8%) | 6876 (83.0%) | 8784 (85.3%) |  | --- | --- | --- | --- |  |
| 1. Divorced | 1155 (7.6%) | 633 (6.8%) | 551 (6.7%) | 634 (6.2%) |  | --- | --- | --- | --- |  |
| 1. Widowed | 564 (3.7%) | 353 (3.8%) | 325 (3.9%) | 339 (3.0%) |  | --- | --- | --- | --- |  |
| Employment |  |  |  |  | <0.001 |  |  |  |  | <0.001 |
| 1. Full time | 7614 (49.9%) | 4549 (48.9%) | 4055 (48.7%) | 5260 (50.9%) |  | 2547 (26.1%) | 1970 (27.6%) | 2196 (29.8%) | 4212 (35.7%) |  |
| 1. Part time | 448 (2.9%) | 288 (3.1%) | 236 (2.8%) | 306 (3.0%) |  | 1623 (16.6%) | 1207 (16.9%) | 1182 (16.0%) | 1960 (16.6%) |  |
| 1. Unemployed | 842 (5.5%) | 425 (4.6%) | 328 (3.9%) | 378 (3.7%) |  | 370 (3.8%) | 272 (3.8%) | 231 (3.1%) | 322 (2.7%) |  |
| 1. Studying | 53 (0.4%) | 33 (0.4%) | 27 (0.3%) | 40 (0.4%) |  | 3927 (40.2%) | 2853 (39.9%) | 3003 (40.7%) | 5219 (35.8%) |  |
| 1. Disability pension | 912 (6.0%) | 478 (5.1%) | 369 (4.4%) | 383 (3.7%) |  | 939 (9.6%) | 587 (8.2%) | 538 (7.3%) | 758 (6.4%) |  |
| 1. Retired | 5377 (35.3%) | 3529 (37.9%) | 3304 (39.7%) | 3957 (38.3%) |  | 371 (3.8%) | 255 (3.6%) | 229 (3.1%) | 330 (2.8%) |  |
| Ever diagnosed Hypertension |  |  |  |  | <0.001 |  |  |  |  | 0.362 |
| 1. No | 12607 (82.7) | 7739 (83.2%) | 6959 (83.7%) | 8653 (83.8%) |  | 9591 (98.1%) | 6992 (97.9%) | 7240 (98.1%) | 11593 (98.2%) |  |
| 1. Yes | 2639 (17.3%) | 1563 (16.8%) | 1360 (16.3%) | 1671 (16.2%) |  | 186 (1.9%) | 152 (2.1%) | 139 (1.9%) | 208 (1.8%) |  |
| Ever diagnosed with Diabetes |  |  |  |  | 0.022 |  |  |  |  | 0.007 |
| 1. No | 14487 (95.0%) | 8851 (95.2%) | 7948 (95.5%) | 9889 (95.8%) |  | 9754 (99.8%) | 7129 (99.8%) | 7365 (99.8%) | 11794 (99.9%) |  |
| 1. Yes | 759 (5.0%) | 451 (4.9%) | 371 (4.5%) | 435 (4.2%) |  | 23 (0.2%) | 15 (0.2%) | 14 (0.2%) | 7 (0.1%) |  |
| Family History of Cancer |  |  |  |  | 0.717 |  |  |  |  | 0.318 |
| 1. No | 8689 (57.0%) | 5246 (56.4%) | 4754 (57.1%) | 5848 (56.6%) |  | 5152 (52.7%) | 3825 (53.5%) | 3906 (52.9%) | 6156 (52.2%) |  |
| 1. Yes | 6557 (43.0%) | 4056 (43.6%) | 3565 (42.9%) | 4476 (43.4%) |  | 4625 (47.3%) | 3319 (46.5%) | 3473 (47.1%) | 5645 (47.8%) |  |
| Energy intake kcal day | 2659 (±930) | 2675 (±920) | 2634 (±886) | 2688 (±854) | <0.001 | 1686 (±575) | 1707 (±593) | 1721 (±567) | 1805 (±544) | <0.001 |
| Food consumption, per day |  |  |  |  |  |  |  |  |  |  |
| 1. Fruits and vegetables ^a^ | 3.26 (±1.9) | 3.67 (±2.3) | 3.87 (±2.5) | 4.73 (±2.8) | <0.001 | 4.15 (±2.3) | 4.55 (±2.7) | 4.89 (±2.9) | 6.05 (±3.2) | <0.001 |
| 1. Wholegrain bread ^a^ | 4.27 (±3.8) | 4.84 (±3.7) | 5.04 (±3.7) | 5.41 (±3.7) | <0.001 | 3.26 (±2.9) | 3.69 (±2.9) | 3.99 (±2.9) | 4.25 (±2.9) | <0.001 |
| 1. Breakfast cereal ^a^ | 0.49 (±0.6) | 0.66 (±0.6) | 0.76 (±0.7) | 0.98 (±0.7) | <0.001 | 0.55 (±0.6) | 0.68 (±0.7) | 0.75 (±0.7) | 0.98 (±0.7) | <0.001 |
| 1. Unprocessed meat ^b^ | 0.55 (±0.4) | 0.48 (±0.4) | 0.43 (±0.3) | 0.40 (±0.3) | <0.001 | 0.52 (±0.4) | 0.44 (±0.4) | 0.40 (±0.3) | 0.37 (±0.3) | <0.001 |
| 1. Processed meat ^b^ | 1.03 (±0.7) | 0.85 (±0.7) | 0.65 (±0.6) | 0.47 (±0.5) | <0.001 | 0.91 (±0.6) | 0.70 (±0.6) | 0.56 (±0.6) | 0.40 (±0.4) | <0.001 |
| 1. Tea ^a^ | 1.05 (±1.2) | 1.14 (±1.4) | 1.16 (±1.6) | 1.28 (±1.7) | <0.001 | 1.02 (±1.1) | 1.06 (±1.3) | 1.15 (±1.8) | 1.24 (±1.6) | <0.001 |
| 1. Coffee ^a^ | 3.53 (±2.4) | 3.61 (±2.3) | 3.63 (±2.2) | 3.53 (±2.0) | <0.001 | 3.07 (±2.3) | 3.23 (±2.1) | 3.24 (±2.0) | 3.19 (±2.0) | <0.001 |
| 1. Soft drinks ^b^ | 1.45 (±1.7) | 1.23 (±1.6) | 1.10 (±1.5) | 0.78 (±1.4) | <0.001 | 1.02 (±1.6) | 0.89 (±1.3) | 0.77 (±1.3) | 0.49 (±1.2) | <0.001 |
| Food consumption per week |  |  |  |  |  |  |  |  |  |  |
| 1. Low fat cheese ^a^ | 0.38 (±1.2) | 0.65 (±1.5) | 0.83 (±1.7) | 1.07 (±1.8) | <0.001 | 0.50 (±1.1) | 0.75 (±1.4) | 0.93 (±1.8) | 1.22 (±1.8) | <0.001 |
| 1. Offal ^b^ | 0.26 (±0.6) | 0.25 (±0.7) | 0.22 (±0.6) | 0.19 (±0.5) | <0.001 | 0.28 (±0.7) | 0.25 (±0.6) | 0.23 (±0.7) | 0.18 (±0.5) | <0.001 |
| 1. Use of olive/canola oil ^a^ | 0.05 (±0.2) | 0.11 (±0.3) | 0.16 (±0.4) | 0.29 (±0.5) | <0.001 | 0.09 (±0.3) | 0.16 (±0.4) | 0.21 (±0.4) | 0.39 (±0.5) | <0.001 |
| 1. Chips ^b^ | 1.80 (±1.8) | 1.65 (±1.7) | 1.52 (±1.8) | 1.34 (±1.7) | <0.001 | 1.49 (±1.8) | 1.34 (±1.7) | 1.16 (±1.5) | 0.98 (±1.4) | <0.001 |
| 1. Chocolate ^a^ | 0.86 (±1.3) | 1.09 (±1.5) | 1.16 (±1.6) | 1.35 (±1.8) | <0.001 | 0.78 (±1.2) | 0.96 (±1.5) | 1.00 (±1.4) | 1.17 (±1.6) | <0.001 |
| 1. Nuts ^a^ | 0.27 (±0.5) | 0.32 (±0.7) | 0.37 (±0.9) | 0.53 (±1.2) | <0.001 | 0.24 (±0.5) | 0.30 (±0.8) | 0.32 (±0.7) | 0.48 (±1.1) | <0.001 |
| 1. Wine ^a^ | 1.19 (±1.7) | 1.54 (±1.9) | 1.75 (±1.9) | 2.28 (±2.0) | <0.001 | 1.20 (±1.6) | 1.43 (±1.7) | 1.63 (±1.7) | 2.15 (±1.9) | <0.001 |
| 1. Beer ^a^ | 5.31 (±7.9) | 5.56 (±7.0) | 5.56 (±6.5) | 5.75 (±5.8) | <0.001 | 1.44 (±3.5) | 1.84 (±3.5) | 2.14 (±3.7) | 2.78 (±4.0) | <0.001 |

^a^ anti-inflammatory
^b^ pro-inflammatory civil status was not
^c^ civil status was not available for women cohort
