# Supplementary material for: Anti-inflammatory Diet Index and Bladder Cancer Risk by Stage: A 22-Year Prospective Swedish Cohort Study (1998–2020)
Source: Cancer Epidemiol Biomarkers Prev. 2026 Mar 31;35(6):1019–26. doi: 10.1158/1055-9965.EPI-25-1733 (PMC13227089; doi:10.1158/1055-9965.EPI-25-1733)
Supplement: Supplementary Table 5 — quantifies the reduction in analytic sample size when restricting to complete cases for alcohol intake and/or physical activity, reporting the remaining number of participants, bladder cancer cases, and person-years, as well as the numbers and percentages excluded due to missing data. Estimates are shown for the overall bladder cancer dataset and for the non-muscle invasive (NMIBC) and muscle invasive (MIBC) stage-specific datasets. [file epi-25-1733_supplementary_table_5_suppst5.docx]

**Supplement Table 5**. Sample reduction when adding alcohol and/or physical activity (complete-case analysis)

| **Dataset** | **Sample** | **N** | **Cases** | **PersonYears** | **Excluded N (%)** | **Excluded cases (%)** | **Excluded PY (%)** |
| --- | --- | --- | --- | --- | --- | --- | --- |
| All BC | Full sample | 79 292 | 1 165 | 1 433 202 | 0 (0.0%) | 0 (0.0%) | 0 (0.0%) |
|  | Alcohol complete-case | 67 138 | 1 056 | 1 238 623 | 12 154 (15.3%) | 109 (9.4%) | 194 579 (13.6%) |
|  | Exercise complete-case | 70 999 | 1 049 | 1 286 020 | 8 293 (10.5%) | 116 (10.0%) | 147 183 (10.3%) |
|  | Alcohol + Exercise complete-case | 60 798 | 956 | 1 120 542 | 18 494 (23.3%) | 209 (17.9%) | 312 660 (21.8%) |
| NMIBC | Full sample | 78 376 | 249 | 1 423 484 | 0 (0.0%) | 0 (0.0%) | 0 (0.0%) |
|  | Alcohol complete-case | 66 313 | 231 | 1 229 775 | 12 063 (15.4%) | 18 (7.2%) | 193 710 (13.6%) |
|  | Exercise complete-case | 70 172 | 222 | 1 277 213 | 8 204 (10.5%) | 27 (10.8%) | 146 271 (10.3%) |
|  | Alcohol + Exercise complete-case | 60 046 | 204 | 1 112 439 | 18 330 (23.4%) | 45 (18.1%) | 311 045 (21.9%) |
| MIBC | Full sample | 78 328 | 201 | 1 422 286 | 0 (0.0%) | 0 (0.0%) | 0 (0.0%) |
|  | Alcohol complete-case | 66 255 | 173 | 1 228 498 | 12 073 (15.4%) | 28 (13.9%) | 193 788 (13.6%) |
|  | Exercise complete-case | 70 133 | 183 | 1 276 200 | 8 195 (10.5%) | 18 (9.0%) | 146 086 (10.3%) |
|  | Alcohol + Exercise complete-case | 60 003 | 161 | 1 111 433 | 18 325 (23.4%) | 40 (19.9%) | 310 852 (21.9%) |
