# Supplementary material for: Anti-inflammatory Diet Index and Bladder Cancer Risk by Stage: A 22-Year Prospective Swedish Cohort Study (1998–2020)
Source: Cancer Epidemiol Biomarkers Prev. 2026 Mar 31;35(6):1019–26. doi: 10.1158/1055-9965.EPI-25-1733 (PMC13227089; doi:10.1158/1055-9965.EPI-25-1733)
Supplement: Supplementary Table 6 — presents sensitivity analyses examining whether additional adjustment for alcohol intake and/or leisure-time physical activity influences the association between the Anti-Inflammatory Diet Index (AIDI) and bladder cancer risk. Hazard ratios (HRs) and 95% confidence intervals are shown for quartiles of AIDI (Q2–Q4 vs Q1) and p-values for trend, using Model 3 for baseline AIDI (1998) and for AIDI modelled as a repeated measure (1998 and 2009; cumulative-average method). Results are reported for the full sample and for complete-case subsets for alcohol, physical activity, and both combined, with models additionally adjusted for total alcohol intake (g/day) and/or leisure-time exercise (hours/week category) as indicated. [file epi-25-1733_supplementary_table_6_suppst6.docx]

**Supplement Table 6**. Sensitivity analyses for additional adjustment for alcohol intake and physical activity: associations between AIDI and bladder cancer risk (Model 3), for baseline (1998) and repeated-measure exposures.

|  |  |  | **Baseline exposure-1998**  **HR (95% CI)** | | | | **Repeated measure of AIDI (1998 & 2009)^¶^ HR (95% CI)** | | | |
| --- | --- | --- | --- | --- | --- | --- | --- | --- | --- | --- |
| **Step** | **Cases** | **PersonYears** | **Q2 vs Q1** | **Q3 vs Q1** | **Q4 vs Q1** | **P for trend** | **Q2 vs Q1** | **Q3 vs Q1** | **Q4 vs Q1** | **P for trend** |
| Full sample (Model 3) | 1 165 | 1 433 202 | 1.00 (0.85, 1.16) | 0.90 (0.76, 1.06) | 0.87 (0.74, 1.02) | 0.049 | 0.97 (0.82, 1.14) | 0.91 (0.78, 1.07) | 0.74 (0.61, 0.89) | 0.002 |
| Alcohol complete-case (Model 3) | 1 056 | 1 238 623 | 1.02 (0.86, 1.20) | 0.88 (0.74, 1.05) | 0.86 (0.73, 1.02) | 0.037 | 0.95 (0.80, 1.14) | 0.92 (0.78, 1.09) | 0.73 (0.60, 0.89) | 0.002 |
| Alcohol complete-case (Model 3 + alcohol) | 1 056 | 1 238 623 | 1.02 (0.87, 1.20) | 0.88 (0.74, 1.05) | 0.86 (0.73, 1.02) | 0.04 | 0.96 (0.80, 1.14) | 0.93 (0.78, 1.10) | 0.73 (0.60, 0.89) | 0.003 |
| Exercise complete-case (Model 3) | 1 049 | 1 286 020 | 1.02 (0.86, 1.20) | 0.92 (0.77, 1.09) | 0.86 (0.72, 1.01) | 0.045 | 0.97 (0.81, 1.15) | 0.91 (0.77, 1.08) | 0.74 (0.61, 0.89) | 0.002 |
| Exercise complete-case (Model 3 + exercise) | 1 049 | 1 286 020 | 1.02 (0.86, 1.20) | 0.92 (0.77, 1.09) | 0.85 (0.72, 1.01) | 0.042 | 0.97 (0.81, 1.15) | 0.91 (0.77, 1.08) | 0.73 (0.60, 0.89) | 0.002 |
| Alcohol+exercise complete-case (Model 3) | 956 | 1 120 542 | 1.03 (0.87, 1.22) | 0.90 (0.75, 1.08) | 0.84 (0.71, 1.00) | 0.026 | 0.93 (0.77, 1.12) | 0.90 (0.76, 1.08) | 0.72 (0.59, 0.88) | 0.002 |
| Alcohol+exercise complete-case (Model 3 + alcohol + exercise) | 956 | 1 120 542 | 1.03 (0.87, 1.22) | 0.90 (0.75, 1.08) | 0.84 (0.71, 1.00) | 0.027 | 0.93 (0.77, 1.12) | 0.91 (0.76, 1.08) | 0.72 (0.58, 0.88) | 0.002 |

Model 3 adjusted for age (stratified), sex, smoking (pack-years), BMI, education, employment status, energy intake (sex-specific centered), diabetes, hypertension, and family history of cancer. + alcohol additionally adjusted for total alcohol intake (g/day). + exercise additionally adjusted for leisure-time exercise (hours/week category; 1997 questionnaire).
