# Supplementary material for: Anti-inflammatory Diet Index and Bladder Cancer Risk by Stage: A 22-Year Prospective Swedish Cohort Study (1998–2020)
Source: Cancer Epidemiol Biomarkers Prev. 2026 Mar 31;35(6):1019–26. doi: 10.1158/1055-9965.EPI-25-1733 (PMC13227089; doi:10.1158/1055-9965.EPI-25-1733)
Supplement: Supplementary Table 7 — shows lagged sensitivity analyses evaluating the association between the Anti-Inflammatory Diet Index (AIDI) and bladder cancer risk after excluding cases diagnosed early in follow-up (no lag, 2-year lag, and 3-year lag). For each lag period, the table reports the number of excluded cases, remaining cases and person-years, hazard ratios (HRs) with 95% confidence intervals for AIDI quartiles (Q2–Q4 vs Q1), and p-values for trend, using fully adjusted Model 3 for baseline AIDI (1998) and for AIDI modelled as a repeated measure (1998 and 2009; cumulative-average method). [file epi-25-1733_supplementary_table_7_suppst7.docx]

**Supplement Table 7**. Lag analyses excluding early bladder cancer cases (fully adjusted Model 3)

|  |  |  |  | **Baseline exposure-1998**  **HR (95% CI)** | | |  | **Repeated measure of AIDI (1998 & 2009)^¶^**  **HR (95% CI)** | | |  |
| --- | --- | --- | --- | --- | --- | --- | --- | --- | --- | --- | --- |
| **Lag** | **Cases excluded** | **Cases** | **Person-years** | **Q2 vs Q1** | **Q3 vs Q1** | **Q4 vs Q1** | **P for trend** | **Q2 vs Q1** | **Q3 vs Q1** | **Q4 vs Q1** | **P for trend** |
| No lag | 0 | 1 165 | 1 433 202 | 1.00 (0.85, 1.16) | 0.90 (0.76, 1.06) | 0.87 (0.74, 1.02) | 0.049 | 0.97 (0.82, 1.14) | 0.91 (0.78, 1.07) | 0.74 (0.61, 0.89) | 0.002 |
| Lag 2 years | 80 | 1 085 | 1 433 127 | 1.00 (0.85, 1.18) | 0.90 (0.76, 1.07) | 0.89 (0.75, 1.04) | 0.092 | 0.99 (0.83, 1.17) | 0.93 (0.79, 1.10) | 0.75 (0.62, 0.91) | 0.004 |
| Lag 3 years | 122 | 1 043 | 1 433 021 | 0.98 (0.83, 1.16) | 0.89 (0.75, 1.06) | 0.87 (0.74, 1.03) | 0.069 | 0.97 (0.81, 1.16) | 0.92 (0.77, 1.09) | 0.74 (0.61, 0.90) | 0.003 |

Model 3 adjusted for age (stratified), sex, smoking (pack-years), BMI, education, employment status, energy intake (sex-specific centered), diabetes, hypertension, and family history of cancer. Lag analyses excluded bladder cancer cases occurring within the first 2 or 3 years after start of follow-up.
