# Supplementary material for: Anti-inflammatory Diet Index and Bladder Cancer Risk by Stage: A 22-Year Prospective Swedish Cohort Study (1998–2020)
Source: Cancer Epidemiol Biomarkers Prev. 2026 Mar 31;35(6):1019–26. doi: 10.1158/1055-9965.EPI-25-1733 (PMC13227089; doi:10.1158/1055-9965.EPI-25-1733)
Supplement: Supplementary Table 8 — reports stratified associations between the Anti-Inflammatory Diet Index (AIDI) and bladder cancer risk by baseline smoking status (never vs ever smokers). Hazard ratios (HRs) and 95% confidence intervals are presented for AIDI quartiles (Q2–Q4 vs Q1) for baseline AIDI (1998) and for AIDI modelled as a repeated measure (1998 and 2009; cumulative-average method), including p-values for trend within each smoking stratum. Fully adjusted Model 3 covariates are specified, and p-values for interaction between AIDI trend and smoking group are provided for both exposure specifications. [file epi-25-1733_supplementary_table_8_suppst8.docx]

**Supplement Table 8**. Stratified analyses by baseline smoking status (never vs ever), fully adjusted Model 3

| **Exposure** | **Category** | **Never smokers HR (95% CI)** | **Ever smokers HR (95% CI)** |
| --- | --- | --- | --- |
| Baseline AIDI (1998) | Q1 | Reference | Reference |
|  | Q2 | 0.69 (0.49, 0.98) | 1.08 (0.91, 1.29) |
|  | Q3 | 0.83 (0.60, 1.15) | 0.91 (0.75, 1.10) |
|  | Q4 | 0.77 (0.57, 1.04) | 0.89 (0.74, 1.07) |
|  | P for trend | 0.15 | 0.12 |
| Repeated AIDI (1998 & 2009)¶ | Q1 | Reference | Reference |
|  | Q2 | 0.87 (0.62, 1.23) | 1.00 (0.83, 1.20) |
|  | Q3 | 0.90 (0.65, 1.25) | 0.91 (0.76, 1.10) |
|  | Q4 | 0.60 (0.41, 0.88) | 0.79 (0.64, 0.97) |
| ¶ | P for trend | 0.017 | 0.021 |

Model 3 adjusted for age (stratified), sex, smoking (pack-years), BMI, education, employment status, energy intake (sex-specific centered), diabetes, hypertension, and family history of cancer. ¶ P for interaction (trend × smoking group): baseline p = 0.911; repeated p = 0.459.
