# Supplementary material for: Anti-inflammatory Diet Index and Bladder Cancer Risk by Stage: A 22-Year Prospective Swedish Cohort Study (1998–2020)
Source: Cancer Epidemiol Biomarkers Prev. 2026 Mar 31;35(6):1019–26. doi: 10.1158/1055-9965.EPI-25-1733 (PMC13227089; doi:10.1158/1055-9965.EPI-25-1733)
Supplement: Supplementary Table 1 — lists the food frequency questionnaire (FFQ)-derived components of the Anti-Inflammatory Diet Index (AIDI), indicates whether each component is classified as anti-inflammatory or pro-inflammatory, specifies the intake cut-offs used to assign a score of 1, and defines the binary scoring rule (1 if the criterion is met; otherwise 0). [file epi-25-1733_supplementary_table_1_suppst1.docx]

**Supplementary Table 1.** Components of the Anti-Inflammatory Diet Index (AIDI), cut-offs, and scoring

| AIDI component (FFQ-derived) | Classification | Criterion to score 1 | Binary score |
| --- | --- | --- | --- |
| Total fruits and vegetables | Anti-inflammatory | ≥6 servings/day | 1 if criterion met; otherwise 0 |
| Tea | Anti-inflammatory | ≥3 servings/day | 1 if criterion met; otherwise 0 |
| Coffee | Anti-inflammatory | ≥2 servings/day | 1 if criterion met; otherwise 0 |
| Wholegrain bread | Anti-inflammatory | ≥2 servings/day | 1 if criterion met; otherwise 0 |
| Breakfast cereal | Anti-inflammatory | ≥1 serving/day | 1 if criterion met; otherwise 0 |
| Low-fat cheese | Anti-inflammatory | ≥1 serving/day | 1 if criterion met; otherwise 0 |
| Olive and canola oil | Anti-inflammatory | >0 servings/day (any consumption) | 1 if criterion met; otherwise 0 |
| Chocolate | Anti-inflammatory | ≥1 serving/day | 1 if criterion met; otherwise 0 |
| Nuts | Anti-inflammatory | ≥2 servings/week | 1 if criterion met; otherwise 0 |
| Red wine | Anti-inflammatory | 2–7 servings/week | 1 if criterion met; otherwise 0 |
| Beer | Anti-inflammatory | 2–14 servings/week | 1 if criterion met; otherwise 0 |
| Unprocessed red meat | Pro-inflammatory* | ≤0.5 servings/day | 1 if criterion met; otherwise 0 |
| Processed red meat | Pro-inflammatory* | ≤0.5 servings/day | 1 if criterion met; otherwise 0 |
| Offal | Pro-inflammatory* | No consumption | 1 if criterion met; otherwise 0 |
| Chips | Pro-inflammatory* | No consumption | 1 if criterion met; otherwise 0 |
| Soft drinks | Pro-inflammatory* | No consumption | 1 if criterion met; otherwise 0 |
